# The Role of the Cephalopod Digestive Gland in the Storage and Detoxification of Marine Pollutants

**DOI:** 10.3389/fphys.2017.00232

**Published:** 2017-04-20

**Authors:** Ana P. Rodrigo, Pedro M. Costa

**Affiliations:** Environmental Toxicology Lab, MARE - Marine and Environmental Sciences Centre, Departamento de Ciências e Engenharia do Ambiente, Faculdade de Ciências e Tecnologia da Universidade Nova de LisboaCaparica, Portugal

**Keywords:** aquatic toxicology, mollusca, Cephalopoda, biomarkers, toxicological pathways, bioaccumulation

## Abstract

The relevance of cephalopods for fisheries and even aquaculture, is raising concerns on the relationship between these molluscs and environmental stressors, from climate change to pollution. However, how these organisms cope with environmental toxicants is far less understood than for other molluscs, especially bivalves, which are frontline models in aquatic toxicology. Although, sharing the same basic body plan, cephalopods hold distinct adaptations, often unique, as they are active predators with high growth and metabolic rates. Most studies on the digestive gland, the analog to the vertebrate liver, focused on metal bioaccumulation and its relation to environmental concentrations, with indication for the involvement of special cellular structures (like *spherulae*) and proteins. Although the functioning of phase I and II enzymes of detoxification in molluscs is controversial, there is evidence for CYP-mediated bioactivation, albeit with lower activity than vertebrates, but this issue needs yet much research. Through novel molecular tools, toxicology-relevant genes and proteins are being unraveled, from metallothioneins to heat-shock proteins and phase II conjugation enzymes, which highlights the importance of increasing genomic annotation as paramount to understand toxicant-specific pathways. However, little is known on how organic toxicants are stored, metabolized and eliminated, albeit some evidence from biomarker approaches, particularly those related to oxidative stress, suggesting that these molluscs' digestive gland is indeed responsive to chemical aggression. Additionally, cause-effect relationships between pollutants and toxicopathic effects are little understood, thus compromising, if not the deployment of these organisms for biomonitoring, at least understanding how they are affected by anthropogenically-induced global change.

## Introduction

Cephalopods are a particular group of invertebrates that share many important features with high-order animals as a result of convergent evolution, with emphasis on nervous system function. These features are, nonetheless, analogs to those of chordates, as cephalopods hold the basic molluscan body plan. Molluscs, however, form a cunningly diverse group of animals, ranging from sedentary filter feeders like bivalves to the giant predator squid *Architeuthis*. Among predators, cephalopods are of special interest in terms of anthropogenic impacts onto food webs, as they feed on a wide range of live prey and have high growth and metabolic rates (see Mangold, 1983), which poses important questions regarding bioaccumulation and tolerance to chemical stressors. However, the physiological and molecular mechanisms underlying toxicopathological effects and detoxification processes in cephalopods are not well understood, albeit the importance of other molluscs, especially bivalves, in biomonitoring and substance testing. Overall, most studies concerning exposure of cephalopods to marine contaminants relate to the accumulation of trace elements, in most cases being limited to commercial species (see Penicaud et al., 2017).

It is suggested that storing metals in various tissues can be an important strategy to cope with metal toxicity (see Miramand and Bentley, 1992; Bustamante et al., 2000; Raimundo et al., 2005 plus the recent review by Penicaud et al., 2017). This efficient strategy, which likely minimizes energetic costs, is seemingly common among Nautiloid and Coleoid cephalopods (Bustamante et al., 2000). However, the mechanisms involved even in these basic processes are not fully resolved, particularly in the case of organic contaminants. This gap noticeable for invertebrates in general, bivalves included, albeit the attention the digestive gland has been receiving as target for bioaccumulation and biomarker analysis for being the analog the vertebrate liver. While there is indication that detoxification and excretion processes may indeed occur with the assistance of specialized cells (Costa et al., 2014), this issue is not entirely consensual as the digestive gland is able to provide long-term storage of both toxic and essential metals, such as Cd and Zn (Bustamante et al., 2002b) The growing level of genomic annotation for bivalves and a few cephalopods also indicates that CYP-like enzymes and respective organic xenobiotic pathways are active in molluscs (Cheah et al., 1995).

As such, the present review aims at summarizing the state-of-the-art on the role of digestive gland in the detoxification of organic and inorganic contaminants in cephalopods, emphasizing the comparative microanatomy, physiology, and molecular processes among various groups of molluscs that, however, indicate that toxicological pathways in cephalopods may be more diverse and complex than anticipated.

## Form and function of the cephalopod digestive gland

**Figure 1:**
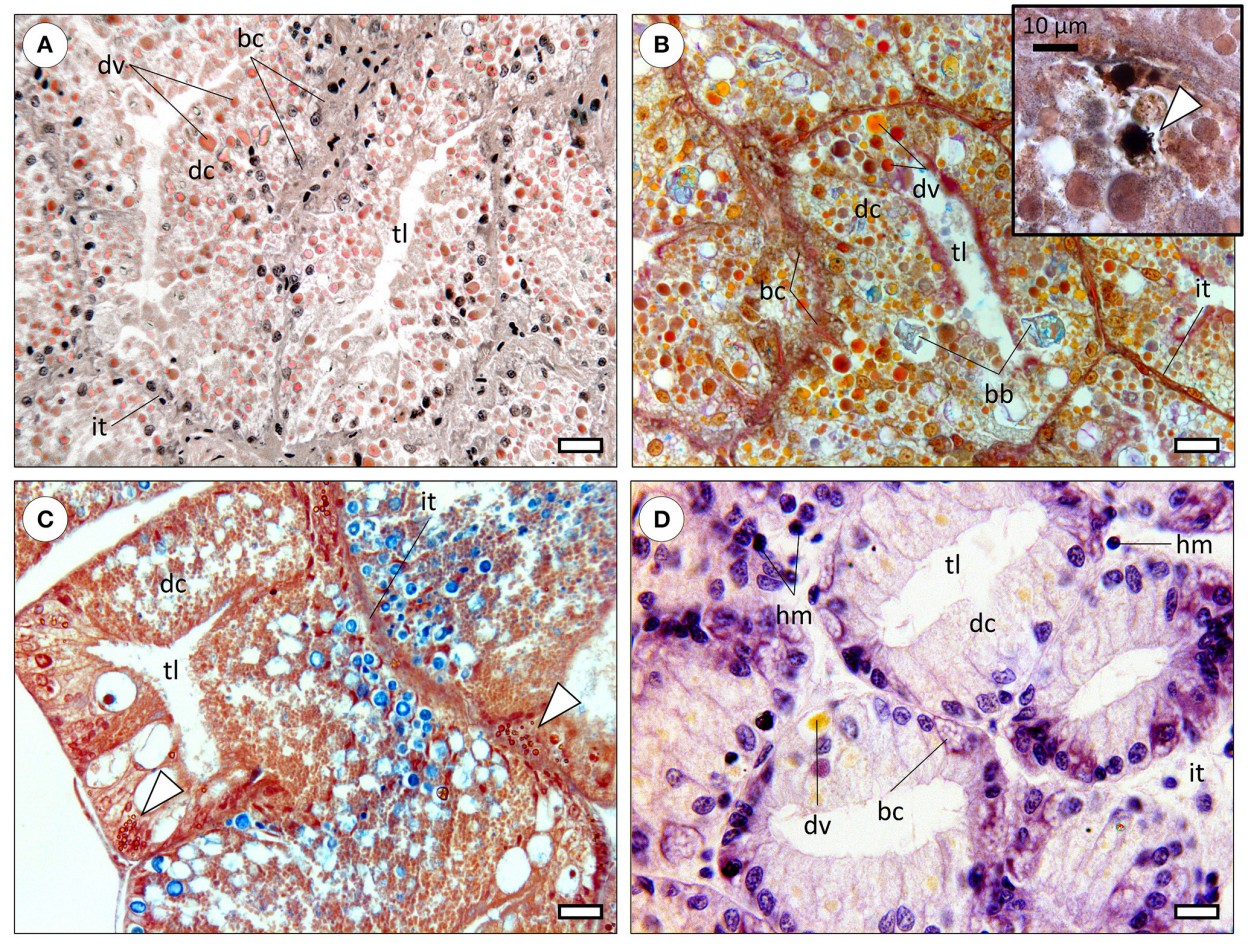
**Comparative histology of the molluscan digestive gland (paraffin sections)**. bc, basal cells (also called replacement; crypt, basophilic or pyramid cells); dc, digestive cells; dv, digestive vacuoles; hm, haemocytes; it, intertubular tissue; tl, tubule lumen. **(A)** Digestive gland of the common octopus (*Octopus vulgaris*) evidencing large digestive tubules (*diverticula*) formed mostly by digestive cells. The distinctive digestive vacuoles of cephalopods are naturally pigmented and traditionally referred by the French term “boules.” Haematoxylin & Eosin. Scale bar: 25 μm. **(B)** Micrograph of the digestive gland of a cuttlefish (*Sepia officinalis*), showing a similar structure to that of *Octopus*. Brown bodies (bb) are distinctive of sepioids, being comprised of amorphous, undigested, materials. Tetrachrome stain. Scale bar: 25 μm. *Inset*: Basal cells were observed to hold calcic *spherulae* that include other metals as well, embedded in a proteinaceous matrix but the issue needs further research. The presence of calcium in *spherulae* in basal cells is here determined histochemically (stained black) through the von Kossa reaction, counterstained with Nuclear Fast Red (arrowhead). **(C)** Section through the digestive gland of the marine gastropod *Onchidella celtica* (Pulmonata), evidencing a similar structure and to that of cephalopods, albeit differences in the histochemical signal of digestive vacuoles, here predominantly blueish (from sugars), likely due to the herbivore feeding regime. The staining is similar to that of the preceding panel. The specimen was fixated in Zenker's solution, which contains (potassium) bichromate that reacts with metallic compounds originating yellow-orange deposits (arrowheads), once again visible in basal cells. Scale bar: 25 μm. **(D)** Section across the digestive gland of a bivalve (*Ruditapes decussata*), stained with Haematoxylin and Eosin. The tubules are smaller than previous examples and digestive cells less intricate with respective to variety, quantity and natural coloration of digestive vacuoles, regardless of digestive phase (which is similar among all panels). Basal cells are again evident and bear vesicular-like structures, potentially *spherulae* or similar. Note the wider and sparser intertubular tissue within which haemocytes can be found, as bivalves have an open circulatory system. Scale bar: 12 μm.

The molluscan digestive gland is a multi-task annex to the digestive tract, involved in secretion of digestive enzymes, extra—and intracellular digestion, substance storage and excretion (Bidder, 1966). As in many invertebrates, organs are called to perform multiple functions due to reduced differentiation comparatively to vertebrates. The basic structure of the digestive gland is well-conserved among molluscs, being formed by blind-end indigitations called “tubules” or more accurately, *diverticula*, being connected to the gut (specifically to the caecum, in cephalopods) by ducts (Budelmann et al., 1997). Refer to Figure 1 for a comparative overview of the molluscan digestive gland. In cephalopods, albeit the lack of an absolute consensus, three distinct cells types have been identified. Digestive cells are the most abundant, followed by basal (replacement) and the more elusive excretory cells, characterized by a single, large hydropic vacuole that may bear mineral precipitates. Note that basal cells are commonly termed crypt and basophilic cells in gastropods and bivalves, respectively. Although demonstrated in cephalopods (e.g., Boucaud-Camou, 1968; Costa et al., 2014), the existence of specialized excretory cells is not consensual in other molluscs and their specific function in cephalopods is not well understood. There are, nonetheless, reports on changes in size and number of hydropic vacuoles of digestive gland cells of gastropods and bivalves as a result of exposure to mixed metallic and organic toxicants (Zaldidar et al., 2007; Lobo et al., 2010).

Cephalopod digestive gland epithelia are more complex than other molluscs' with respect to specialized endosomes. In fact, structures such as “boules” (vacuoles involved in digestion and enzyme secretion) and “brown bodies” (excretion of crystalline salts and amorphous materials) are seemingly exclusive. The lack of detailed studies integrating digestive gland histology and cytology with molecular pathways, as well as the lack of comparative studies between molluscan taxa hinders understanding how molluscs evolved to handle hazardous substances.

Accumulation of toxicants in the digestive gland depends on their mechanisms of apical entry. Bustamante et al. (2002b) revealed that Cd and Zn enter the cephalopods' digestive gland directly *via* food and indirectly *via* blood, in the latter case if uptake occurs from seawater. Nonetheless, the same authors disclosed that the elimination of these metals is faster if uptaken through water. It must be noted that the existence of a closed circulatory system in these molluscs, likely render the organ particularly efficient for nutrient absorption and as a filtering system for peripheral fluids. Indeed, unlike bivalves for instance, the cephalopod digestive gland possesses an intricate network of arteriole-like blood vessels (e.g., Swift et al., 2005; Costa et al., 2014). To these features is added the ability to form (and eventually release) mineral corpuscles called *spherulae* (spherocrystals) in the basal cells, first noticed by Martoja and Marcaillou (1993) and more recently described by Costa et al. (2014) which may thus have an important role in metal homeostasis, similarly to what has been suggested for some gastropods (Volland et al., 2012).

The first descriptions of the microstructure of the cephalopod digestive gland are almost as old as histology itself (refer to the pioneer works by Frenzel, 1886 and Cuénot, 1907). These were complemented by important histochemical descriptions being made from the 1960s onward that favored structural and digestion-related aspects (e.g., Boucaud-Camou, 1968; Semmens, 2002; Martinez et al., 2011). However, even for bivalves, which are the most investigated invertebrates by toxicologists, there are many gaps about the relation between form and function of the digestive gland and toxicant metabolism.

## The digestive gland in bioaccumulation and detoxification of metals

The vast majority of literature focusing on toxicants in cephalopods relates to metals, as cephalopods are known to bioaccumulate impressive amounts of hazardous elements, like Cd, albeit others, such as Hg, appearing to be less significant, which indicates metal- and organ-specific pathways (Penicaud et al., 2017). It has been shown that the digestive gland holds the highest concentrations of essential (like Cu and Zn) and non-essential (such as Ag, Cd and Pb) metals in several cephalopods, with emphasis on *Sepia* and *Octopus*, comparatively to other organs, mantle and arms included, the latter of which raise particular concerns regarding human consumption (e.g., Raimundo et al., 2004, 2005; Seixas et al., 2005; Bustamante et al., 2006; Pereira et al., 2009). However, the digestive gland has been identified as the main metal accumulation organ even in the Nautiloidea (Pernice et al., 2009). Available data suggests that the cephalopod digestive gland is particularly efficient in the retention of these elements, likely as a function of chelating agents, especially proteins. In fact, there have been a few works that related concentrations of metals in this organ with different types of largely undisclosed proteins distributed through several subcellular partitions. For instance, Raimundo et al. (2010a) noticed that, in the common octopus, Pb was associated to unknown high molecular weight proteins while Zn, Cu and Cd showed high affinity to both high and low molecular weight proteins. Somewhat similar associations were recorded in the digestive glands of red arrow squid (*Nototodarus gouldi*) by Finger and Smith (1987) and in the common cuttlefish by Bustamante et al. (2006). These authors suggested that these associations may relate to different metal detoxification mechanisms, in many cases likely modulated by metal burden *per se*, through the induction of chelating proteins. Interestingly, Costa et al. (2014), demonstrated histochemically that the metal-containing *spherulae* in cuttlefish digestive gland basal cells are formed by a matrix of proteinaceous materials, being released into the lumen of tubules as cells differentiate. However, there seems to be some selectivity in the accumulation of metals in *spherulae*. Unlike Cu, which appears to be accumulated mostly in these structures, Fe accumulates essentially in the cytosol of digestive cells, which has been confirmed histochemically in the independent works by Martoja and Marcaillou (1993) and Costa et al. (2014) with *Sepia officinalis*. Altogether, basal cell *spherulae* may be common in molluscan digestive glands, even though their role and formation is not well understood. Volland et al. (2012), for instance, demonstrated the existence of these structures in basal cells in the marine gastropod *Strombus*, albeit being seemingly not involved in Cd bioaccumulation upon induced exposure. In agreement, Bustamante et al. (2002a) reported that, in several different cephalopods, Cd is mostly cytosolic.

It has also been suggested that metallothioneins (MTs), well described in bivalves, play an important role in the formation of *spherulae* (Martoja and Marcaillou, 1993). However, in cephalopods, MT induction in the digestive gland may not be entirely consistent with exposure to metals, even in the case of exposure to strong inducers such as Cd (Bustamante et al., 2002a; Raimundo et al., 2010b; Rodrigo et al., 2013). It is possible that elements such as Cd, are not involved in detoxification via *spherulae* (thus remaining in the cytoplasmic fraction), which in its turn, relies on MT and, potentially, unknown high molecular weight proteins (see Penicaud et al., 2017, for a summary). This interesting perspective, indicates that there can be novel mechanisms of toxicity and detoxification of non-essential metals like Cd in cephalopods that need to be unraveled. As such, the known mechanisms for MT expression, described essentially for vertebrates, namely those relying on metallothionein transcription factor (MTF) mediation may not apply entirely or at all. In Table 1 are summarized the most relevant among the very few publications on biomarker responses in cephalopod digestive gland in an ecotoxicological context, which includes also the MT response as potential indicator of exposure to metals.

**Table 1:** **A compilation of different biomarkers and other indicators of exposure to environmental toxicants measured in cephalopods**.

**Species**	**Approach**	**Target**	**Biomarker responses**	**Substances of interest**	**Geographical area**	**Main outcomes**	**Reference**
*Sepia officinalis*	*In vitro* bioassay	Digestive gland cells	Enzyme activity (trypsin, collagenase, hyaluronidase and pronase) plus cell viability	Cu, Zn, Ag	U.K.	Ag can be highly toxic as it inhibited digestive enzymes, whereas Zn only caused adverse effects at higher concentrations. Cu had no significant effects.	Le Bihan et al., 2004
*Sepia officinalis*	Field sampling	Adults (digestive gland and gills)	LPO, GST, GSHt, GSH/GSSG, MT	Mixed environmental toxicants (metallic and organic)	Portugal	Lipid peroxidation was one of the most significant biomarkers in the digestive gland, together with GST. Gills probably more sensitive.	Rodrigo et al., 2013
*Sepia officinalis*	Bioassay	Juvenile (whole-organism sampling)	LPO, GST, SOD, Catalase, MT	Ag, As, Cd, Co, Cr, Cu, Fe, Mn, Ni, Pb, Se, V, Zn and Zn alone	France	Maternal transfer of metals in the cuttlefish. Evidence for deficient Zn regulation.	Le Pabic et al., 2015
*Sepia elegans* *Sepia officinalis* *Sepia orbignyana* *Loligo vulgaris* *Illex coindetii* *Todarodes sagittatus* *Eledone cirrhosa*	Field sampling	Adults (digestive gland)	MT	Cd	France (Bay of Biscay) and Faroe Islands.	No differences between size and gender were noted. Cadmium was mainly located in the soluble fraction of digestive gland cells, probably bound to cytosolic proteins. Limited participation of these proteins in the detoxification of Cd.	Bustamante et al., 2002a
*Octopus pallidus*	Bioassay	Adults (digestive gland)	CYP, EROD, ECOD, NADPH cytochrome c oxidase	β-naphthoflavone, Aroclor 1254	Australia	Cytochrome P450 appears to be inducible by Aroclor 1254, as measured by ECOD activity and CYP quantitation.	Cheah et al., 1995
*Octopus vulgaris*	Field sampling	Adults (various tissues, including the digestive gland)	MT	Mixed environmental metals	Portugal	Apparent MT-Cd and MT-Cr associations in digestive gland. No such association was obtained between Co, Ni and As in analyzed tissues.	Raimundo et al., 2010b
*Octopus vulgaris*	Field sampling	Adults (multiple organs, including the digestive gland)	DNA-SB (comet assay)	Zn, Cu, Cd and Pb	Portugal	No significant differences effect of animal size. DNA damage could be associated to Cd but was too high in digestive gland for clear associations.	Raimundo et al., 2010a
*Octopus vulgaris*	Field sampling	Adults (arms and digestive gland)	CAT, SOD, GST, LPO, PCO	Cu, Zn, Pb, Cd, As	Portugal	Effective anti-oxidant pathways in the digestive gland, including the activity of GST as a scavenger for lipid hydroperoxide radicals.	Semedo et al., 2012
*Octopus vulgaris*	Field sampling	Adults (arms and digestive gland)	EROD, ECOD	PAHs	Portugal	Low levels of PAHs in the digestive gland and low levels of EROD and ECOD activities.	Semedo et al., 2014
*Octopus vulgaris*	Bioassay	Paralarvae (whole organism)	CAT, GST, HSP70 and SOD gene expression (by qRT-PCR)	Mn, Cd	Spain	Gene expression suggests that Cd has an oxidative stress potential greater than Mg with hazardous effects and mortality at relatively low concentrations, triggering stress-related responses as defense.	Nicosia et al., 2015

*CAT, catalase activity; CYP, cytochrome P450; DNA-SB, DNA strand breakage; ECOD, ethoxycoumarin O-deethylase; EROD, ethoxyresorufin O-deethylase; GSH, glutathione (reduced); GSSG, glutathione (oxidized); GST, glutathione S-transferase activity; HSP70, heat-shock protein 70 KDa; LPO, lipid peroxidation; MT, metallothionein induction; NADPH, nicotinamide dinucleotide (reduced); PAH, polycyclic aromatic hydrocarbon; PCO, protein carbonyl content; SOD, superoxide dismutase activity; qRT-PCR, quantitative real-time polymerase chain reaction*.

## Evidence for the metabolism of organic toxicants

Although far less common than for metals, some studies addressed the issue of bioaccumulation of various organic hazardous substances in the cephalopod digestive gland, from polycyclic aromatic hydrocarbons (PAHs) and polychlorinated biphenyls (PCBs) to amnesic shellfish toxin (Costa et al., 2005, 2009; Danis et al., 2005; Storelli et al., 2006; Semedo et al., 2014). Nevertheless, the pathways of detoxification and elimination of organic substances, pollutants and toxins (endogenous or exogenous) that are called bioactive compounds are strikingly more complicated than metal chelation and expression of chelators. These mechanisms have been described mostly for mammals and are not consensual for invertebrates, once again indicating important knowledge gaps, in spite of the relevance of molluscs for ecotoxicologists.

The pathways for drug metabolism, meaning phases I (biotransformation) and II (conjugation), to which is now added a phase III (elimination) have been described in the vertebrate liver (see Ferreira et al., 2014, and references therein). Their functioning in invertebrates is not entirely consensual, in part due to the differential response of common biomarkers between vertebrates and invertebrates. For instance, one of the most important systems involved in phase I, the cytochrome P450 (CYP) monooxygenase complex (involved in the detoxification of many bioactive xenobiotics), has long been shown to hold similarities between invertebrates and vertebrates using cDNA probes and Western blotting techniques (Livingstone, 1994). This includes evidence that at least some octopodid cephalopods express CYP isoenzymes, albeit at reduced levels comparatively to vertebrates (Cheah et al., 1995). The CYP systems are localized mainly in the microsomes of the digestive gland cells of molluscs, although it was also found in other tissues like gills and even in haemocytes (Oehlmann and Schulte-Oehlmann, 2003). Still, the relevance of CYP1A in molluscs (involved in the metabolism of important PAHs, polychlorinated biphenyls and dioxins, as examples) has been disputed through works with bivalves, in favor of other forms, such as CYP4 (Chaty et al., 2004). In contradiction, there is some evidence for CYP1A activity and induction in terrestrial molluscs exposed to toxicants (Snyder, 2000, for a review). It is also important to mention that the pathways leading to increased CYP expression, usually involving xenobiotic-activated nuclear receptors (XANRs), are not well understood in invertebrates (see Richter and Fidler, 2014). In fact, the cephalopod equivalent for the aryl hydrocarbon receptor (Ahr) pathway, which is responsible for increased expression of CYP1A by PAHs and similar in vertebrates, remains a mystery. Still, there are promising findings with bivalves regarding its expression in gills and digestive glands exposed to Ahr agonists like benzo[a]pyrene (e.g., Châtel et al., 2012).

Schlenk and Buhler (1988) suggested that CYPs are more important in the metabolism of endogenous substrates rather than xenobiotic metabolism in Polyplacophora digestive glands, whilst other works evidenced some induction in bivalves (concerning enzyme content and activity) by agents ranging from pharmaceuticals to acrylamide, although unable to metabolize compounds that can be biotransformed in vertebrates or induced in a reduced extent (see for instance Galli et al., 1988; Larguinho et al., 2014). Also, Cheah et al. (1995) disclosed modest induction of CYPs in *Octupus pallidus* digestive gland after exposure to known inducers like β-naphtoflavone and Aroclor, with increased activity of ethoxycoumarin-*O*-deethylase (ECOD) but not for ethoxyresorufin *O*-deethylase (EROD), which is one of the best accepted biomarkers of exposure to bioactive pollutants in vertebrates. This finding is in agreement with the work by Schlenk and Buhler (1988) with chitons, even though Semedo et al. (2014) found negligible activities of both enzymes in wild *Octopus vulgaris*.

The activity of phase II enzymes, such as glutathione *S-transferase* or UDP-glucosyltransferase have been found in cephalopod digestive gland, with evidence that they can be induced by environmental toxicants but their specificity toward a specific class of substances has yet to be demonstrated (Schlenk and Buhler, 1988; Rodrigo et al., 2013; Le Pabic et al., 2015), since phase II enzymes can be induced by many factors, oxidative stress in particular. Inclusively, they were found to be promising targets in *Octopus* paralarvae exposed to metals (Nicosia et al., 2015). Altogether, less specific biomarkers, therefore responsive unspecific physiological challenge, yield more promising outcomes in the cephalopod digestive gland, among which are included superoxide dismutase (SOD) and catalase activities or glutathione induction (Semedo et al., 2012; Rodrigo et al., 2013; Nicosia et al., 2015). Note, though, that there are indications that the activity of oxidative stress enzymes can be significantly modulated by age in cephalopods (Zielinski and Pörtner, 2000). Interestingly, Oellermann et al. (2012) found that cuttlefish may adjust cardiac mitochondrial metabolism *per se* to adjust to thermal challenge, which indicates the ability to fine tune mitochondria-mediated oxidative functions under stress. This may explain the relevance of oxidative stress biomarkers in these animals, despite the missing link between mitochondrial function and toxicant-induced oxidative stress.

Another interesting target could be heat-shock proteins (HSPs) and similar chaperones that interfere with gene expression pathways. They have been described at the transcriptome level in several molluscs, including a few cephalopods (reviewed by Wang et al., 2013), and can be pointed as promising biomarkers (refer also to Oehlmann and Schulte-Oehlmann, 2003). In spite of the absence of similar work on cephalopods, with the exception of the work by Nicosia et al. (2015) with *Octopus* paralarvae (whose transcript levels increased with Cd exposure concentration), HSP70 induction has provided interesting results in other molluscs (bivalves in particular) subjected to several environmental stressors, from bacterial infection to metals, PAHs, pharmaceuticals, and biotoxins (Köhler et al., 1996; Boutet et al., 2003; Cellura et al., 2006; Song et al., 2006; Mello et al., 2012; Gust et al., 2013).

Altogether, detoxification of organic toxicants in the cephalopod digestive gland does occur, albeit potentially better adapted to dispose of toxins and unwanted by-products from feed and basal metabolism. It must also be considered that endpoints that are common biomarkers in vertebrates may not be the most adequate in molluscs, with particular respect to phase I. At the present, responses related to phase II and oxidative stress appear to be more viable biomarkers of exposure to bioactive organic toxicants, together with ECOD activity (refer to Table 1). Phase III of detoxification is still a subject of debate, being essentially reported for vertebrates, fish included. The action of specific transporters, like ATP-binding cassette (ABC) transporters, located in digestive tract cells, is described for the elimination of xenobiotic metabolites (reviewed by Ferreira et al., 2014). Even though not yet scrutinized in cephalopods, ABC transporters have been investigated in other molluscs and their action in gills during metal-induced challenge has already been reported (e.g., Della Torre et al., 2015). It would be of relevance to attempt to relate form and function of excretory vacuoles in digestive gland cells with phase III activity. Additionally, while the lack of genomic annotation is a clear drawback to unravel potentially novel mechanisms of detoxification through phases I to III in cephalopods, with the advent of state-of-the-art proteomics, metabolomics, (epi)genomics and transcriptomics tools (next-generation sequencing of genomic DNAs and RNAs), plus bioinformatics, more mechanistic information could be retrieved regarding detoxification pathways in cephalopods and other molluscs while assisting biomarker discovery.

## Concluding remarks

Even though it remains to be seen whether cephalopods can have such a significant role in biomonitoring as bivalves, the importance of these animals in ecosystem functioning, fisheries and more recently aquaculture dictates the relevance of investigating how these animals cope with anthropogenic pressures than endanger their habitats. The still scarce literature on physiological and molecular pathways related to detoxification of noxious substances suggests, however, that the digestive gland plays a major role, much in similarity with the vertebrate liver. As genomic annotation for these animals is slowly coupled with traditional biomarkers approaches that until recently were considered more or less exclusive to vertebrates, it may now be inferred that the toxicological pathways in cephalopods are more complex than expected for both metallic and organic xenobiotics, steering toward a new direction to understand the adaptative mechanisms of cephalopods to impacted marine ecosystems.

## Author contributions

AR and PC are responsible for the conceptual design and writing of the manuscript. PC supervised the work.

### Conflict of interest statement

The authors declare that the research was conducted in the absence of any commercial or financial relationships that could be construed as a potential conflict of interest.
